# Flow Cytometric Analysis: Four-Year Experience in a Tertiary Care Centre of Pakistan

**DOI:** 10.7759/cureus.764

**Published:** 2016-09-01

**Authors:** Imran N Ahmad, Salman Assad, Muhammad Rahman, Haider Ghazanfar

**Affiliations:** 1 Shifa International Hospital, Shifa College Of Medicine, Islamabad, Pakistan; 2 Department of Neurology & Neurosurgery, Shifa Tameer-e-Millat University, Islamabad, Pakistan; 3 Student, Shifa College of Medicine, Islamabad, Pakistan; 4 Department of Pathology, Shifa College Of Medicine, Islamabad, Pakistan

**Keywords:** flow cytometric analysis, hematolymphoid malignancies, immunopathology, pakistan

## Abstract

Purpose:

This study summarizes a four-year experience from the analysis of hematolymphoid malignancies in Pakistani population using a database of six-colored flow cytometry.

Methods:

A cross-sectional survey of 323 specimens of hematolymphoid malignancies using six-colored flow cytometry (FC) was carried out in Shifa International Hospital, Islamabad, Pakistan from June 2012 to June 2016. The criterion for specimen adequacy was that the cases have abnormal populations by FC, and the specimen age (time from biopsy to being examined by the six-color FC tube) of three days or less was to be included in the study. Clinical follow-up of greater than six months was required for a negative flow cytometric study without a subsequent biopsy. Data analysis was done using  Statistical Package for the Social Sciences (SPSS) version 21. One-way analysis of variance (ANOVA) was used to compare diagnosis with some antibodies used.

Results:

The number of specimen within certain age groups included were: 0-15 years; 111 (34.3%), 16-30 years; 65 (20.12%), 31-45 years; 47 (14.5%), 46-60 years; 46 (14.2%) and ≥ 60 years; 54 (16.7%). Hematological malignancies were documented in descending order of sequence with B-cell acute lymphoblastic leukemia (27.9%), acute myeloid leukemia (26.3%), chronic lymphocytic leukemia (13.3%), T cell acute lymphoblastic leukemia (7.7%), non-Hodgkin's lymphomas (5%), hairy cell leukemia (1.9%), chronic myeloid leukemia (0.3%), paroxysmal nocturnal hemoglobinuria (0.6%) and plasma cell dyscrasias (0.6%). The mean number of antibodies used were 12.68 ± 2.97. One-way ANOVA was used to compare diagnosis with some antibodies used. Statistical significance was found between diagnosis and number of antibodies used (F= 5.23 p<0.001).

Conclusion:

B cell acute lymphoblastic leukemia is most commonly diagnosed at tertiary care units in Pakistan using six-colored flow cytometry. Adoption of these complicated techniques has reinforced the need for optimization and further enhancement of flow cytometric procedures.

## Introduction

The flow cytometry (FC) is a technology where normal and abnormal components of cells in a tissue are detected. Undoubtedly, it has produced fruitful results when various samples of patients with hematolymphoid malignancies are analyzed. A quick assessment of the physical characteristics of complex samples from peripheral blood, body fluids and tissues can be done with extra and intracellular immune phenotypes of individual cells [1-2]. As such, flow cytometry has proven to be an essential tool for detection and characterization of malignant cells in a background of normal cellular components. This information is valuable in the diagnosis of patients with a variety of hematolymphoid malignancies as well as for providing data relevant to prognosis and patient management. The aim of flow cytometry is to collect data on the intrinsic (structure size or complexity) and extrinsic (nucleic acid content, antigenic makeup or functional attributes) characteristics of cells. During flow cytometry, the cells present in suspension pass one by one, in rapid succession through one or more monochromatic light sources (lasers). As each cell passes through the laser light, it scatters the incident light. The conditions in which flow cytometry is indicated includes an increased leukocyte count (eosinophilia, lymphocytosis, monocytosis), the presence of blasts or atypical cells in the peripheral blood, body fluids or bone marrow (cytopenias, especially bi-cytopenia and pancytopenia), plasmacytosis or monoclonal gammopathy and organomegaly or enlargement of tissue masses. In these clinical situations, flow cytometric immunophenotyping (FCI) emerges as a useful screening tool to differentiate between neoplastic and non-neoplastic conditions. Also, it is a necessary technology to stage an already-diagnosed hematolymphoid neoplasm, to monitor the response to treatment that also involves the detection of minimal residual disease (MRD) [3].

Flow cytometry plays an integral role in the field of organ transplantation, detection of anti-HLA antibodies and cross-matching [4-5]. FC has replaced the Ham acidified serum and sucrose lysis tests for laboratory paroxysmal nocturnal hemoglobinuria (PNH). This disorder is due to an acquired defect in the PIG-A gene in hematopoietic stem cells, resulting in a deficiency of the glycosylphosphatidylinositol (GPI) molecule that anchors many proteins, including CD55 and CD59, to the lipids in the cell membrane. Since the defect is in the stem cell, it would include RBCs, granulocytes, and platelets, and would not be able to express GPI-anchored proteins. As the defect is acquired and not inherited, only a fraction of the total hematopoietic cells is affected. Neoplasm of mature-T and natural killer (NK) cells can be identified by using flow cytometric immunophenotyping through the detection of aberrant antigen expression [6-7]. Diagnosis of plasma cell disorders is based on increased serum or urine gamma globulins and can be divided into polyclonal and monoclonal gammopathies. The monoclonal gammopathies can be subdivided into monoclonal gammopathy of undetermined significance (MGUS) and overt plasma cell dyscrasias which include plasmacytoma, plasma cell myeloma with variants, plasma cell leukemia, amyloidosis, immunoglobulin serious and light chain diseases. The diagnosis of plasma cell dyscrasias usually requires demonstration of an abnormal phenotype or clonality, increased plasma cells (greater than 10% of marrow cells) and classification based on combinations of clinical, morphologic, laboratory and radiologic findings.

FCI is a useful tool for the identification of abnormal plasma cells. It also helps to distinguish between lymphoid and plasma cell neoplasms. Furthermore, flow cytometric testing may provide additional prognostic information [8-10]. Classical Hodgkin's lymphoma (CHL) is characterized by large, multinucleate Hodgkin and Reed-Sternberg (HRS) cells comprising one percent or less of the lymph node cells in an infiltrate of reactive cells such as histiocytes, eosinophils, lymphocytes and plasma cells [11].Historically CHL could not be immunophenotyped by FC, but recent studies show that CHL can be diagnosed by FC [12-13]. The most appropriate example simultaneous analysis of multiple characteristics by flow cytometry is the immunophenotyping of leukemias and lymphomas. Although it is tough to classify most acute myeloid leukemias by phenotype alone, flow cytometry can aid in distinguishing certain types of acute myeloid leukemias, such as acute promyelocytic leukemia [14-15]. Therapy-resistant leukemias can also be detected by FC [16]. In ALL (acute lymphocytic leukemia), the phenotype has been shown to correlate strongly with outcome [17]. The current study was conducted in a tertiary care hospital to analyze patients with various hematolymphoid malignancies by using six to eight colored flow cytometry.

## Materials and methods

This is a cross-sectional analysis of 323 specimens of hematolymphoid malignancies diagnosed with six to eight colored flow cytometry from February 2012 to June 2016 at Shifa International Hospital Islamabad, Pakistan. Morphologic diagnoses were determined by a combination of histological examination, conventional FC, and immunohistochemistry at flow cytometry laboratory. Criteria for specimen adequacy were as follows: (a) Cases have abnormal populations by FC. (b) All tissues without abnormal FC population have the presence of greater than 50,000 events. (c) Specimen age (time from biopsy to being evaluated by the six-color FC tube) of three days or less to be included in the study. Age groups included as: 0-15 years: 111 (34.3%), 16-30 years: 65 (20.12%) ; 31-45 years: 47 (14.5%) ; 46-60 years: 46 (14.2%);  ≥ 60 years: 54 (16.7%). Out of 323 specimens, 210 (65%) were from males, and 113 (35%) were taken from females (Table 1). Some antibodies used as compared to the age groups is shown in Table 2. Samples were collected from bone marrow 149/323 (46.1%), blood 172/323 (53.2%), lymph node 2/323 (0.61%).

**Table 1:** Gender Distribution

Gender	Number (n = 323)	Percentage (%)
Male	210	65
Female	113	35

**Table 2:** Number of Antibodies Used for Certain Age Groups

Patient Age	Mean Number of Antibodies Used
0-15	12.13 ± 2.85
16- 30	12.46 ± 3.12
31-45	13.77 ± 2.87
46-60	12.85 ± 2.96
≥60	14 ± 13.02

### Preparation of specimens

**Figure 1:**
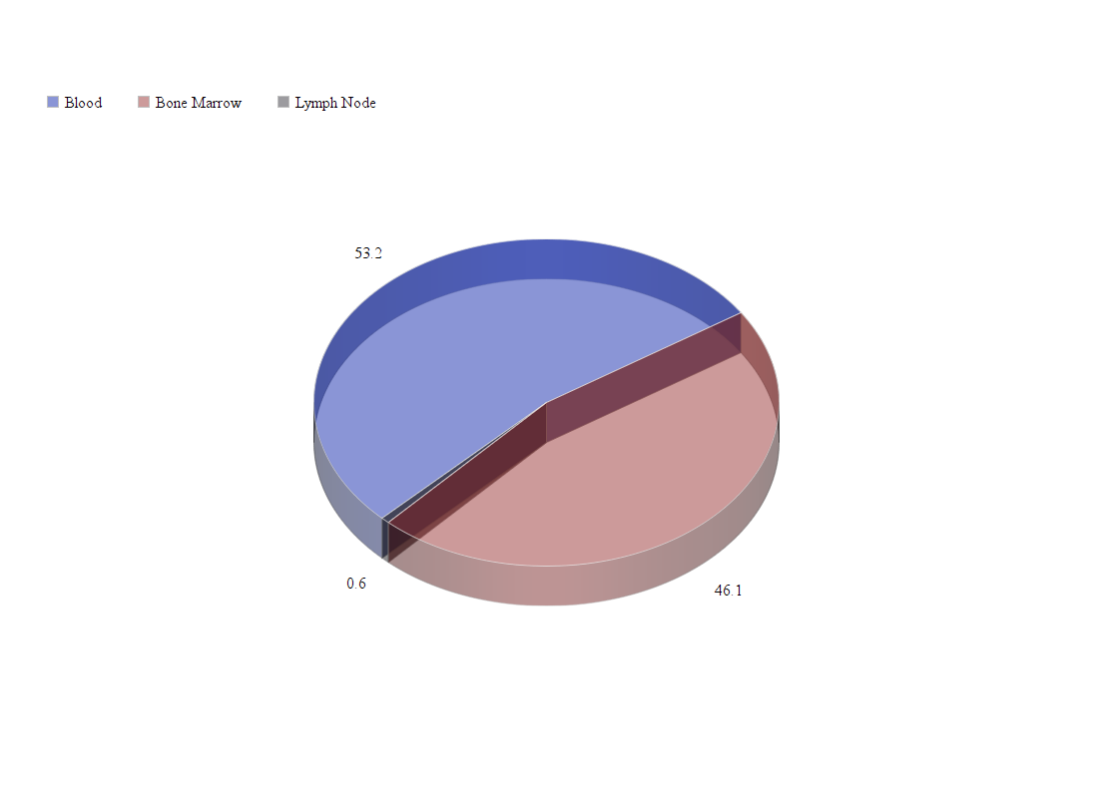
Specimens Taken The pie chart indicates percentages (%) of specimens taken for diagnosis of maligancies through flow cytometry.

Specimens included in the study were taken from blood, bone marrow and lymph nodes (Figure 1).

### Lymph node

Samples taken from fine needle aspiration (FNAs) were performed under ultrasound guidance by a radiologist or, alternatively, at the bedside by a cytopathologist. Twenty-two to twenty-five gauge needles with ten milliliter syringes were used for FNA samples; on the other hand, samples obtained by radiologists were often done without aspiration. Two passes per sample were arranged to ensure adequate material for FC analysis. By using a modified Wright-Giemsa stain, all FNA samples on the site were assessed by a cytopathologist and on the basis of clinical judgment and morphologic abnormalities, the pathologist decides whether the material is to be sent for FC analysis or not. RPMI Medium (Roswell Park Memorial Institute Medium) was used for material and sent instantly for flow cytometric analysis to immune-pathology. FC reports and FNA smears were done independently without information of the diagnosis regarding the other. FC is initially examined independently of the FNA smears. The FC report is created by non-cytopathologists and merged with the cytology report, allowing for retrospective independent evaluation of FC. The final cytologic diagnosis integrates cytomorphology with FC results.

### Peripheral blood

Diagnostic phenotypic information can be analyzed by peripheral blood. The analysis on the population of interest is done by FC. Circulating malignant cells estimation is provided by white blood count. Generally, collection volume has to be adjusted because cells of interest in blood are in lesser numbers in contrast to bone marrow. A sodium heparin tube was used to collect 10-20 ml (minimum of 5 x 106 cell yield) and a sodium heparin plus Ethylenediaminetetraacetic acid (EDTA) tube was utilized for flow immunophenotyping. Smear and complete blood count results were given with specimens for flow immunophenotyping.

### Bone marrow aspirate

Diluted citrate solution may be used if aspiration syringe needs the internal pre-coating to avoid clotting. For immunophenotyping, internal pre-coating of specimens can be done to prevent clotting as results can be compromised due to clots being hardly visible which prevents an optimal immunophenotyping. Specimens with 2 to 3 ml aseptically aspirated bone marrow smear in sodium with a minimum of 5 x 106 cell yield along with complete blood count were sent for immunophenotyping. Cold packs were utilized for transport, and the specimens were put in the refrigerator.

### Fluorescently labeled antibodies and controls

Coulter (BC; Hialeah, FL) or BD was utilized for the fluorescently labeled antibodies. Antibodies used in the single six-color FC tube for all the specimens (antibody clone designation and supplier) included CD64-FITC (22; BC), CD30-PE (Ber- H83; BD), CD40-PE-Cy5.5 (MAB89; BC), CD20-PE-Cy7 (B9E9; BC), CD95-APC (DX2; BD), and CD3-APC-Cy7 (SK7; BD) or CD3-APC-H7 (SK7; BD). To determine background fluorescence, an appropriate fluorescence minus one control was also utilized. Extensive lymphoma panel was used for FC immunophenotyping which included the following antibodies: CD8-fluorescein isothiocyanate (FITC)/CD4-phycoerythrin (PE)/CD3-Texas Red (ECD)/CD7-PE cyanine 5 (PC5), FITC/CD19-PE/CD10-ECD/CD5- PC5, FITC/CD19-PE/CD10-ECD/CD5-PC5,FMC7-FITC/CD103-PE/CD20-ECD/CD19-PC5,CD38-FITC/CD23-PE/CD19-ECD/CD138-PC5,andCD16+CD56-FITC/CD25-PE/ CD3-ECD/CD14-PC5; all tubes contained CD45-PE cyanine 7 (PC7).The panel was altered, if necessary, based on clinical data, limited material, concurrent morphologic assessment, or previously known lymphoma subtype. Clinical follow-up of greater than six months was required for a negative flow cytometric study without a subsequent biopsy. The data analysis was done using Statistical Package for the Social Sciences (SPSS) version 21. One-way ANOVA was used to compare diagnosis with a number of antibodies used.

## Results

**Figure 2:**
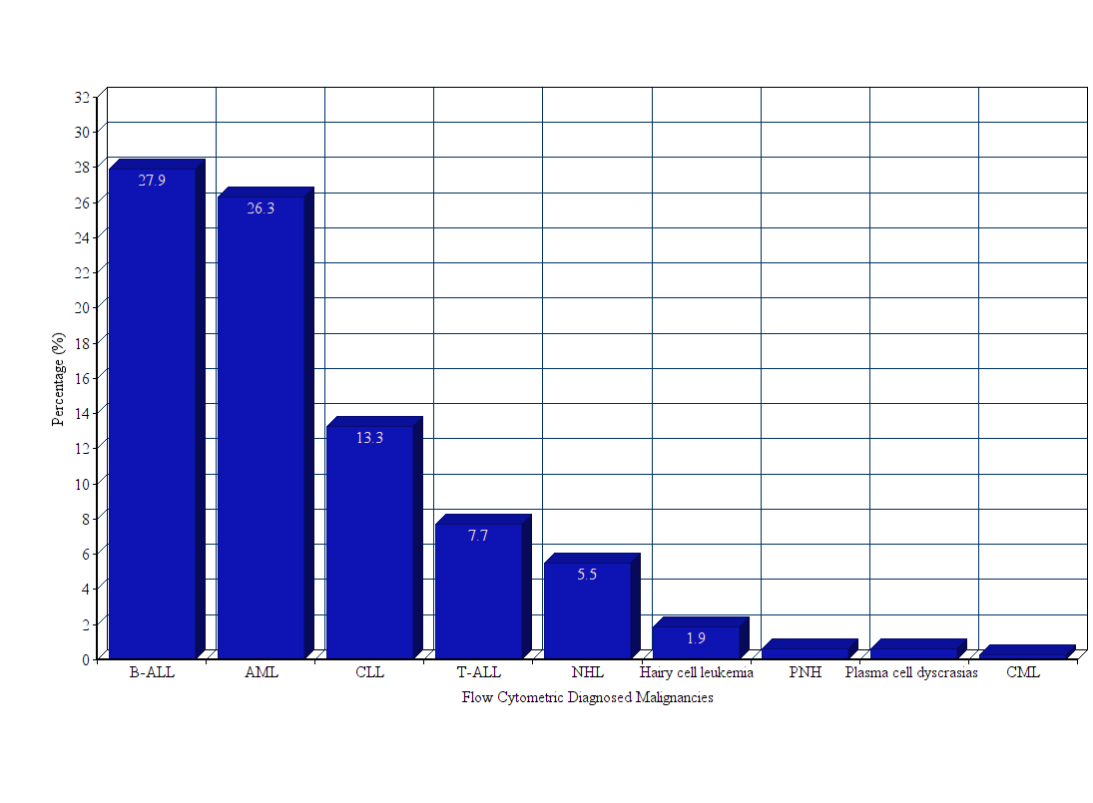
Flow Cytometric Diagnosed Maligancies B cell acute lymphoblastic leukemia (B-ALL), T cell acute lymphoblastic leukemia (T-ALL), acute myeloid leukemia (AML), chronic myeloid leukemia (CML), chronic lymphocytic leukemia (CLL), paroxysmal nocturnal hemoglobinuria (PNH), non-Hodgkin's lymphomas (NHL)

The hemato-lymphoid malignant cases which were also morphologically confirmed demonstrated demographic features expected of these malignancies. The total sum of 323 cases of various hematolymphoid malignancies was seen in our laboratory. A number of specimens with the following age groups were included: 0-15 years; 111 (34.3%), 16-30 years; 65 (20.12%), 31-45 years; 47 (14.5%), 46-60 years; 46 (14.2%), ≥ 60 years; 54 (16.7%); depicted in Table 1. Out of 323 specimens, 210 (65%) were taken from males and 113 (35%) were taken from females. B-cell acute lymphoblastic leukemia (B-ALL) 90 (27.9%), acute myeloid leukemia (AML) 85 (26.3%), chronic lymphocytic leukemia (CLL) 43 (13.3%), T cell acute lymphoblastic leukemia (T-ALL) 25 (7.7%), non-Hodgkin's lymphomas (NHLs) 16 (5%), hairy cell leukemia 6 (1.9%) paroxysmal nocturnal hemoglobinuria (PNH) 2 (0.6%), plasma cell dyscrasias 2 (0.6%), chronic myeloid leukemia (CML) 1 (0.3%) were found as common hematolymphoid malignancies (Figure 2).

The remaining samples 21 (6.5%) showed no malignancy, marrow in remission 18 (5.6%), no residual diseases 13 (4%) and diluted sample 1 (0.3%). The mean number of antibodies used were 12.68±2.97 as shown in Table 3. One-way ANOVA was used to compare diagnosis with some antibodies used. Statistical significance was found between diagnosis and number of antibodies used (F= 5.23 p<0.001). Clinically used terms like marrow in remission indicate the improved outcomes of diseases after the appropriate management. Likewise, no malignancy depicts that there is no detection of malignant cells in a sample, and no residual disease indicates that there is no malignant component after required therapy.

**Table 3:** Number of Cases Diagnosed After Flow Cytometry B-cell acute lymphoblastic leukemia (B-ALL), T-cell acute lymphoblastic leukemia (T-ALL), acute myeloid leukemia (AML), chronic myeloid leukemia (CML), chronic lymphocytic leukemia (CLL), paroxysmal nocturnal hemoglobinuria (PNH), non-hodgkin lymphomas (NHL)

Diagnosis	Frequency	Mean± Standard Deviation Number of Antibodies Used	
B-ALL	90 (27.9%)	12.43±2.36	
AML	85 (26.3%)	14.15±3.58	
CLL	43 (13.3%),	13.42±3.28	
T-ALL	25 (7.7%)	13.16±2.23	
NHL	16 (5%)	13.25±2.43	
Hairy Cell Leukemia	6 (1.9%)	12.17±1.17	
PNH	2 (0.6%)	7.00±2.97	
Plasma Cell Dyscrasias	2 (0.6%)	11.50±0.707	
CML	1 (0.3%)	16	
No malignancy	21 (6.5%)	10.67±3.85	
No residual disease	13 (4.0%)	12.31±2.39	
Marrow In Remission	18 (5.6%)	12.94±2.46	

We evaluated 323 specimens to determine the diagnostic utility of the novel six-color assay for the detection of the neoplastic cells blinded to any clinical, morphologic or immunophenotypic information, so as to provide an unbiased evaluation of this assay. The blood, bone marrow, and lymph node specimens included 323 cases including in a variety of reactive cases, B and T cell non-Hodgkin's lymphomas, non-hematopoietic neoplasms and some neoplasms that can be confused morphologically with chronic Hodgkin's lymphoma (CHL). They are diffused large B-cell lymphoma [DLBCL], nodular lymphocyte-predominant Hodgkin's lymphoma [NLPHL] and anaplastic large cell lymphoma.

## Discussion

Individual cells in suspension are assessed for the presence and absence of specific antigens (phenotype) by flow cytometric immunophenotyping. In the evaluation of hematologic malignancies, several measures are adopted in the interpretation and application of this immunophenotypic information: (a) detection of cells from different lineages and recognition of whether they are mature or immature; (b) finding abnormal cells through recognition of antigen expression that varies significantly from normal cells; (c) thorough records of the phenotype of abnormal cell populations (for example for the absence or presence of the particular antigens), in comparison to their normal cell counterpart, a documentation of the increased or decreased intensity of staining by antibodies labeled by fluorochrome; (d) evaluation of whether the information is diagnostic of a distinct disease, and if not, then development of a list of possible entities with proposition of additional studies such as immunohistochemistry that might be of diagnostic value; (e) provision of immunophenotypic information that might be of additional prognostic value, and including the information that will help in the identification of targets for potential directed therapy. Whenever a specimen is received for flow cytometric testing, preference is made regarding the cell antigens and lineages to be assessed. The decision of FC evaluation for a specimen is based on the type of the sample, the clinical indication for testing listed on the requisition, morphologic findings, history of prior flow cytometric testing, clinical history, results of other laboratory testing, and perhaps results of any preliminary screening tests done in the flow cytometric laboratory [4].

According to the medical indications recognized by the 2006 Bethesda group, an agreement was reached on the cell lineages and the antigens which should both be evaluated and included in the primary assessment of each lineage [4]. Using this approach, flow cytometric immunophenotyping of clinical specimens can offer a rapid screening for hematologic neoplasms and play a significant role in diagnosis and classification. Malignancies of mature lymphoid cells include non-Hodgkin lymphoma and chronic leukemia lymphoid neoplasms. Such diseases are recognized by immunophenotypes that are identical to normal mature lymphoid cells (e.g. surface immunoglobulin on mature B cells) and lack of antigenic features of immaturity such as expression of TdT, CD34, or weak intensity staining for CD45.Through recognition of lineage-associated antigens and mature lymphoid cells, malignancies can be separated into those of B cell, T cell, and NK cell lineages. Multicolor flow cytometric approach can be used to identify the abnormal cells with a characteristic phenotype in a majority of the patients with Hodgkin lymphoma. Despite this fact, FC technique has not yet been adopted by our laboratory or most of the laboratories worldwide [5].

Flow cytometric immunophenotyping studies are crucial for the diagnosis of mature B cell lymphoid neoplasms through the detection of phenotypically abnormal cells which belongs to the B cell lineages and the recognition of phenotypes characteristic of distinct disease entities. FC can also be used to identify the expression of targets for potential antibody-directed therapy and provide additional prognostic information such as CD38 and ZAP-70 expression in chronic lymphocytic leukemia/small lymphocytic lymphoma (CLL/SLL) [6]. FC is becoming an established method for the evaluation of minimal residual disease after therapy [6-7]. Clinically used terms like marrow in remission indicate the improved outcomes of diseases after the appropriate management. Likewise, no malignancy shows that there is no detection of malignant cells in a sample, and no residual disease indicates that there is no malignant component after required therapy. Mature T and NK cell malignancies can often be recognized by flow cytometric immunophenotyping through detection of aberrant antigen expression [18]. Also, aberrant antigen expression by neoplastic T and NK cells must be distinguished from the normal phenotypic variations which are seen between the multiple subsets of non-neoplastic cells. Although flow cytometric immunophenotyping is a useful tool for the identification and distinction of plasma cell from mature B-lymphoid malignancies with plasmacytic differentiation, its diagnostic utility is limited by difficulties faced in enumerating plasma cells [19-20]. Even using sensitive techniques, flow cytometric immunophenotyping recognizes fewer plasma cells than the paraffin section of biopsy sections [21].

### Limitations

This is single-center cross-sectional survey based on the Pakistani population. The results of this analysis cannot be generalized to other geographical regions of the world. A number of clinical parameters used to diagnose a malignancy were excluded that we believe would have added more resilience to statistical analysis and outcome of the survey.

### Implications of the study

The study implies upon the researchers to look for reasons of most documented B cell neoplasm in the geographical distribution of Pakistan and innovate the diagnostic tools to exclude other rarely reported hematolymphoid malignancies. The survey will help oncologists from Pakistan to consider B cell neoplasm as a foremost provisional diagnosis in patients presenting with suspected malignancy.

## Conclusions

The survey clearly manifests the most common demographic presentation of the B cell acute lymphoblastic leukemia (B-ALL) 27.9% and indicates that the second most reported hematolymphoid malignancy is acute myeloid leukemia (AML) 26.3% at the tertiary care unit in Pakistan. In the past ten years, clinical flow cytometry has evolved from a technique that was primarily used to characterize large populations of malignant cells to one that can routinely evaluate small populations of cells for subtle aberrancies in antigen expression. Adoption of these more complicated techniques has reinforced the need for optimization and further enhancement of flow cytometric procedures and for the interpretation of the results to be done by individuals who are familiar with all aspects of the testing that may affect the quality of the data. It is of utmost importance that the interpreters of the flow cytometric data have an extensive and adequate knowledge about the phenotypes of diverse and healthy cell populations, can recognize deviations from the normal, and can discuss and apprehend the clinical significance of the flow cytometric findings.
